# Decreasing Compensatory Ability of Concentric Ventricular Hypertrophy in Aortic-Banded Rat Hearts

**DOI:** 10.3389/fphys.2018.00037

**Published:** 2018-02-23

**Authors:** Alexandre Lewalle, Sander Land, Eric Carruth, Lawrence R. Frank, Pablo Lamata, Jeffrey H. Omens, Andrew D. McCulloch, Steven A. Niederer, Nicolas P. Smith

**Affiliations:** ^1^Department of Biomedical Engineering, King's College London, St. Thomas's Hospital, London, United Kingdom; ^2^Department of Bioengineering, University of California, San Diego, San Diego, CA, United States; ^3^Radiology Department, University of California, San Diego, San Diego, CA, United States; ^4^Department of Medicine, University of California, San Diego, La Jolla, CA, United States; ^5^Faculty of Engineering, University of Auckland, Auckland, New Zealand

**Keywords:** ejection fraction, hypertrophy, cardiac compensation, aortic banding, left ventricle, computational modeling

## Abstract

The cardiac system compensates for variations in physiological and pathophysiological conditions through a dynamic remodeling at the organ, tissue, and intracellular levels in order to maintain function. However, on longer time scales following the onset of ventricular pressure overload, such remodeling may begin to inhibit physiological function and ultimately lead to heart failure. This progression from compensatory to decompensatory behavior is poorly understood, in particular owing to the absence of a unified perspective of the concomitantly remodeling subsystems. To address this issue, the present study investigates the evolution of compensatory mechanisms, in response to overload, by integrating diffusion-tensor MRI, echocardiography, and intracellular and hemodynamic measurements within consistent computational simulations of aortic-banded rat hearts. This approach allows a comparison of the relative leverage of different cardiac properties (geometry, passive mechanical stiffness, fiber configuration, diastolic and peak calcium concentrations, calcium-binding affinity, and aortic impedance) to affect cardiac contraction. Measurements indicate that, following aortic banding, an ejection fraction (EF) of 75% was maintained, relative to control rats, despite significant remodeling of the left-ventricular wall thickness (increasing by ~90% over 4 weeks). Applying our framework, we identified the left-ventricular wall thickness (concentric hypertrophy) and the intracellular calcium dynamics as playing the dominant roles in preserving EF acutely, whereas the significance of hypertrophy decreased subsequently. This trend suggests an increasing reliance on intracellular mechanisms (average increase ~50%), rather than on anatomical features (average decrease ~60%), to achieve compensation of pump function in the early phase of heart failure.

## 1. Introduction

The heart is a dynamic system that adapts to changes in external loading conditions to maintain its physiological function. In cases of ventricular pressure overload, where the ejection of blood from the ventricle is hindered by elevated aortic pressure, the maintenance of this function requires an increase in the generated stress to maintain systemic blood flow (Liu et al., 2010). In practice, this maintenance is achieved through a remodeling of cardiac properties across cellular, tissue, and organ scales. The initial maintenance of function, with no externally observable symptoms of heart failure, is commonly described as cardiac compensation. On a longer time scale, however, the initial benefits of remodeling often transition to dysfunction, suggesting that the remodeling responses have either collectively lost their effectiveness or progressed to a point where they have become detrimental to cardiac function (Bregagnollo et al., 2007; Patten and Hall-Porter, 2009). This so-called decompensatory stage ultimately leads to heart failure. Despite its clinical importance, the details of the underlying mechanisms and chronology of their evolution remain poorly understood. Specifically, while isolated manifestations of the overall response to pressure overload have been extensively documented (Okoshi et al., 2004; Patten and Hall-Porter, 2009; Chemaly et al., 2012), they still provide only a piecewise picture of compensation that remains to be unified.

Pressure overload typically arises in cases of aortic-valve stenosis, a relatively common pathology affecting more than 10% of the elderly, in which the aortic-valve opening is narrowed, thereby increasing aortic resistance (Osnabrugge et al., 2013). The characteristic symptoms of this condition are typically observed in aortic-banded (AB) rats and mice, common experimental models where the aorta is artificially constricted to impede blood flow (Doering et al., 1988; Siri et al., 1989; Doggrell and Brown, 1998; Patten and Hall-Porter, 2009; DeAlmeida et al., 2010). The constriction can be inserted at various sites of the aorta: ascending, transverse or descending, but in all cases, the resulting pressure overload induces significant remodeling (Molinari et al., 2016). Over a time scale of weeks, the cardiac tissue mass increases and the diastolic and systolic left ventricle (LV) diameters are reduced, while cardiac contractility and ejection performance are preserved (Litwin et al., 1995; Okoshi et al., 2004; Takimoto et al., 2005; Bregagnollo et al., 2007; Chemaly et al., 2012). This ventricular hypertrophy, characterized by a thickening of the muscular wall, allows the heart to generate the higher pressure required to maintain blood flow. Concomitantly, the collagen content in the cardiac interstitium typically increases, which may contribute to altering the tissue mechanical properties (Doering et al., 1988; Jalil et al., 1989; Hu et al., 2003; Takimoto et al., 2005). At the intracellular level, evolving gene expression impacts on metabolism, calcium handling, and contractility (Keung, 1989; Nordin et al., 1989; Feldman et al., 1993). The relative contributions of these separate changes to overall compensatory behavior may themselves evolve over time as the heart gradually approaches decompensation.

To better understand this evolution of the compensated phase toward decompensation, this study investigates cardiac remodeling in compensated aortic-banded rats. By comparing these rats with sham-operated controls (i.e., without aortic banding), we seek to isolate the impact of AB on the cardiac system, to effectively probe two different stages of compensation. For each case, we integrate echocardiography, diffusion-tensor MRI (DT-MRI), hemodynamic, and calcium-stimulus measurements into computational models designed to quantify and compare different contributions to compensation. We then apply an *in-silico* sensitivity analysis to quantify the “compensatory ability” of individual model parameters in the vicinity of the *in vivo* conditions.

The AB rat hearts used in this study display concentric hypertrophy, when compared to the control (sham-operated) rats, without however showing symptoms of decompensation or heart failure. An additional feature is that, despite the magnitude of the imposed change of physiological conditions, the overall cardiac performance, as measured by the left-ventricular ejection fraction EF (the ratio of the stroke volume SV to the end-diastolic volume EDV), remains remarkably constant. This constancy in EF, together with the absence of symptoms of decompensation, underpins our characterization of this phase as a phenomenological manifestation of compensation, i.e., before the onset of decompensation. Having replicated the measured principal features of the cardiac cycle using the computational models, we calculated and compared the partial sensitivities of EF to the functional parameters of each heart model. We thus interpret these sensitivities as reflecting the overall capacity of the heart to respond dynamically under pathologically induced stress to maintain normal function. Taken as a whole, this computational modeling approach provides a theoretical framework for unifying the experimental datasets, which conceptually represent separate time points in the evolution of compensation.

## 2. Methods

### 2.1. Analysis framework

**Figure 1:**
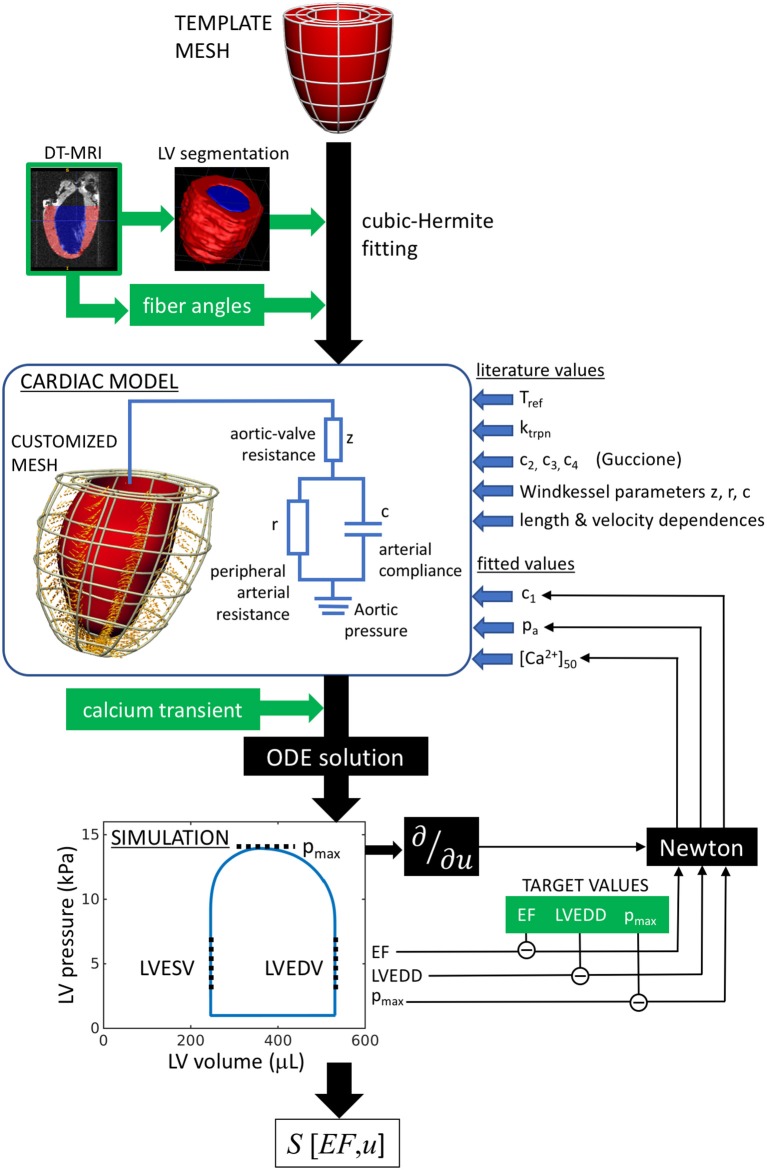
Analysis workflow, as described in section 2.1. A template mesh is customized by making maximal use of available data derived from the rat heart. The green labels denote data measured within this study. The solutions of the ODEs are compared with echo (EF, LVEDD) and hemodynamic measurements (*p*_max_), to complete the model parameterization using a Newton method. The chosen free fitting parameters are *c*_1_, *p*_a_, and [Ca^2+^]_50_.

The workflow of the model characterization is outlined in Figure 1. In summary, the cardiac model is customized to an individual rat heart by first fitting a generic template mesh to a segmented three-dimensional DT-MRI image. The primary eigenvectors of the diffusion tensor at each image voxel specify the fiber directions, which are fitted and incorporated into the mesh. Contraction is then stimulated by applying an intracellular calcium transient simultaneously and ubiquitously throughout the muscle tissue. The numerical solution of the ordinary differential equations describing the system mechanics yields the pressure-volume characteristics over a full heart beat, that can be compared directly to hemodynamic and echocardiographic measurements performed on the individual rats. The tissue stiffness, aortic pressure, and calcium-binding sensitivity are then tuned to achieve maximal consistency with the rat-specific measurements.

In some instances, it was not possible to reproduce the measured diastolic and contractile properties using any choice of model parameters because the diameter of the segmented heart was too similar to the measured end-diastolic diameter. An analysis of these hearts indicated the likely presence of residual LV pressure during the *ex vivo* fixation process, performed prior to DT-MRI, resulting in an overestimate of the LV dimensions in diastasis, and hence precluding reliable model parameterization. Those hearts were therefore discarded. In total, one SHAM heart and three AB hearts were successfully simulated and considered for further analysis.

Having thus parameterized the heart models, we investigated the impact of aortic banding on cardiac function. Conceptually, our methodological approach associates the remodeling process with a hypothetical trajectory through a multidimensional parameter space *U* with coordinates that describe the cardiac system:

(1) $$u=\{\tilde{c_{1}},{\left[{\text{Ca}}^{2+}\right]}_{\text{50}},p_{\text{a}},z,\text{PCa},\text{DCa},u_{\text{geom}},u_{\text{fiber}}\},$$

where $\tilde{c_{1}}$ measures the passive tissue stiffness, [Ca^2+^]_50_ is the calcium-binding sensitivity of troponin, *p*_a_ is the aortic pressure, *z* is the aortic-valve impedance, PCa and DCa are respectively the peak and diastolic intracellular calcium concentrations, and *u*_geom_ and *u*_fiber_ are generalized coordinates representing the geometrical and fiber configurations. From this perspective, the end points of the trajectory represent the real heart models (i.e., the individual SHAM and AB hearts), while the trajectory represents the continuous evolution from a “healthy” heart (SHAM) to the remodeled heart, as a result of aortic banding. At each intermediate state considered along the trajectory, a new hypothetical heart model was fitted again using the process outlined in Figure 1 and assuming linearly interpolated values for the phenotypes.

The geometrical and fiber parameters *u*_geom_ and *u*_fiber_, which represent the geometrical and fiber structures, are defined as continuous variables bounded by $u_{\text{geom}}^{\left(\text{SHAM}\right)}=u_{\text{fiber}}^{\left(\text{SHAM}\right)}=0$ and $u_{\text{geom}}^{\left(\text{AB}\right)}=u_{\text{fiber}}^{\left(\text{AB}\right)}=1$. Other points along the trajectory are expressed by intermediate values of *u*_geom_ and *u*_fiber_, representing hypothetical heart structures generated by linear interpolation of, respectively, the node coordinates or the fiber angles of the end-point models.

Within this formalism, we quantified the “compensatory ability” of a given model, with respect to each variable, in terms of the sensitivity of EF, considered as a function over *U*:

(2) $$S[\text{EF},u]=\frac{\partial \text{EF}(u)}{\partial u}\cdot \frac{u^{\left(\text{AB}\right)}-u^{\left(\text{SHAM}\right)}}{\text{EF}(u)}.$$

This formulation ensures the non-dimensionality of *S* and considers changes in a given parameter *u* in proportion to the phase-space distance spanned by the overall trajectory. The derivatives ∂EF(*u*)/∂*u* were calculated by applying changes to *u* that were sufficiently small to remain in the local linear regime around the model considered. As discussed below, we interpret *S*[EF, *u*] as a measure of the ability of each parameter *u* to control EF and maintain the heart in a compensated state.

### 2.2. Rats

An initial cohort of 20 Sprague-Dawley rats was initially considered for echocardiography (University of California, San Diego). Eight weeks (±4 days) after birth, 10 of the rats were applied an AB on the transverse aorta with a 0.5 mm clip, while the other 10 underwent a sham surgery. All animal use followed NIH guidelines and was approved by the Institutional Animal Care and Use Committee (IACUC) at the University of California, San Diego.

The heart rate of each rat was monitored every 7 days post intervention. Echocardiograms were measured immediately prior to the operation, and subsequently after 2 and 4 weeks. The rats were rested on an IACUC-approved heating pad and anesthetized with 1.5% isoflurane in 100% O_2_ by nose cone. M-mode echocardiographic images were acquired using a GE vivd/I imaging system. The entire procedure took approximately 15–20 min per animal. The animals were transferred to their cages and quickly regained full consciousness.

Four weeks post operation, the rats underwent a hemodynamic study to determine the maximum aortic pressure during LV ejection using a manometer-tipped catheter (Millar, Houston, TX, US). A subset of six rats from the initial cohort were then imaged by diffusion-tensor MRI (DT-MRI). Two of the SHAM and four of the AB hearts were extracted and fixed in diastasis by arrest using a high-potassium solution, followed immediately by fixation to approximate the relaxed state. Following a method described elsewhere (Benson et al., 2011), the muscle-fiber orientations were determined from DT-MRI measurements performed on these fixed hearts.

### 2.3. Ventricular dimensions and ejection fraction estimation

**Figure 2:**
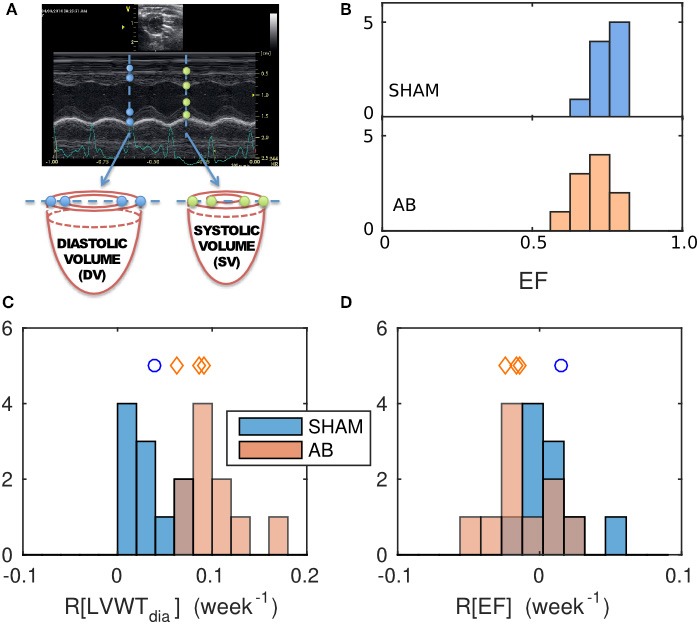
Geometrical characterization and its evolution, determined from echocardiography data and comparison between the SHAM and AB hearts. **(A)** Sample M-mode echograph, showing the time course of the LV inner- and outer-wall boundaries. The data can be used to estimate the ejection fraction EF = 1 − (systolic volume)/(diastolic volume), deduced by considering the deformation of a cylindrical basal slice of the left ventricle, as shown. **(B)** Histograms of EF values obtained for the SHAM (mean = 0.75, s.d. = 0.05, *n* = 10) and AB hearts (mean = 0.71, s.d. = 0.06, *n* = 10) 4 weeks post intervention. Average relative rates of change of geometrical features were calculated from echo measurements done at 0, 2, and 4 weeks post-intervention, for a geometrical feature *y*, defined as *R*[*y*] = 〈*dy*/*dt*〉/*y*(*t* = 0), with **(C)** *y* = diastolic LV wall thickness LVWT_dia_ (SHAM mean = 0.03/week, s.d. = 0.02/week, *n* = 10; AB mean = 0.095/week, s.d. = 0.030/week, *n* = 10) and **(D)** *y* = EF (SHAM mean = 0.008/week, s.d. = 0.022/week, *n* = 10; AB mean = −0.013/week, s.d. = 0.022/week, *n* = 10). The blue- and red-shaded histograms correspond to the SHAM and AB heart datasets, respectively. The blue circle and the red diamond symbols indicate the LVWT_dia_ and EF values of the individual SHAM and AB hearts used in the computational simulations (Table 1).

M-mode echocardiograms were measured *in vivo* along the vertical diameter of a basal slice, displaying the time course of the LV inner and outer walls over five heart beats (Figure 2A). Mean diastolic and systolic LV diameters (LVD) and wall thicknesses (LVWT), were determined for each rat at time points *t* = 0, 2, and 4 weeks from the AB or SHAM intervention. To assess the potential impact of aortic banding, the average rate of change of these parameters *y*, over the 4-week period, was quantified using the metric

(3) $$R[y]=\frac{1}{y(t=0)}\cdot {\langle \frac{dy}{dt}\rangle }_{\text{4 weeks}}.$$

The EF for each heart was estimated by treating the M-mode echocardiography images as cross sections through a cylindrical LV slice (Figure 2A). The change in the internal volume of these cylinders reflects the ventricular EF in so far as the blood ejected from the basal region of the LV constitutes the majority of the total ejection. The incompressibility of the wall tissue implies that any change in the cross-sectional area of the cavity wall is accompanied by an extension of the cylinder height *h* in the base-apex direction to conserve the tissue volume π(*r* + LVWT)^2^*h* − π*r*^2^*h*, where *r* = LVD/2. Hence,

(4) $$\begin{array}{l} \text{EF}=1-\frac{\text{systolic volume}}{\text{diastolic volume}}\\ \;\approx 1-\frac{\pi r_{\text{sys}}^{2}h_{\text{sys}}}{\pi r_{\text{dia}}^{2}h_{\text{dia}}}\\ \;=1-{\left(\frac{r_{\text{sys}}}{r_{\text{dia}}}\right)}^{2}\cdot \frac{{\left(r_{\text{dia}}+{\text{LVWT}}_{\text{dia}}\right)}^{2}-r_{\text{dia}}^{2}}{{\left(r_{\text{sys}}+{\text{LVWT}}_{\text{sys}}\right)}^{2}-r_{\text{dia}}^{2}}. \end{array}$$

To validate this approach, we compared the result of Equation (4) with a more explicit measurement of the rat LV volume using MRI data available in the literature (Wise et al., 1998). The good agreement in the two approaches confirmed the suitability of Equation (4) for our present purpose. The details of this analysis are presented in Appendix 1.

### 2.4. DT-MRI

Diffusion-weighted images were acquired *ex vivo* in 15 non-collinear directions on a 7 T scanner equipped with Avance II hardware (Bruker) using a DTI-EPI sequence. A gradient insert allowed for a 1 T/m maximum gradient strength and maximum slew rate of 11,250 T/m/s. Imaging parameters were as follows: FOV 20 × 20 × 20 mm, isotropic 200 μm resolution, gradient duration 2 ms, gradient separation 11 ms, nominal *b*-value 500 s/mm^2^, number of non-diffusion weighted images 1. The total scan time was approximately 1 h. This scan protocol was repeated five times and the resultant images were manually averaged in AFNI (NIH, Bethesda, MD).

The resulting image stacks were segmented (ITK-SNAP) and used to reproduce the LV geometry and muscle-fiber configuration in the computational model, as explained below.

### 2.5. Calcium transients

We complemented the above imaging results with measurements of intracellular calcium stimuli, performed in separate experiments at physiological temperature (37°C) in single myocytes derived from sham and aortic-banded Wistar rat hearts 6 weeks following the the placing of a constriction on the ascending aorta (Oslo University, Norway) (Røe et al., 2017). Samples from the aortic-banded rats required that the hearts display wall thickening in excess of 1.9 mm with no signs of systolic heart failure (left atrial diameter less than 5.0 mm and no left ventricular dilation). The overall phenotypes of the animals were similar to those described in section 2.2. The EF was preserved in the AB rats, compared to SHAM-operated rats, consistent with the rats in section 2.2.

LV myocytes were isolated using a standard enzymatic dispersion technique (Louch et al., 2011). Calcium measurements and calibrations were done as described by Røe et al. (2017).

### 2.6. Finite-element model and simulation

#### 2.6.1. Geometrical mesh

**Figure 3:**
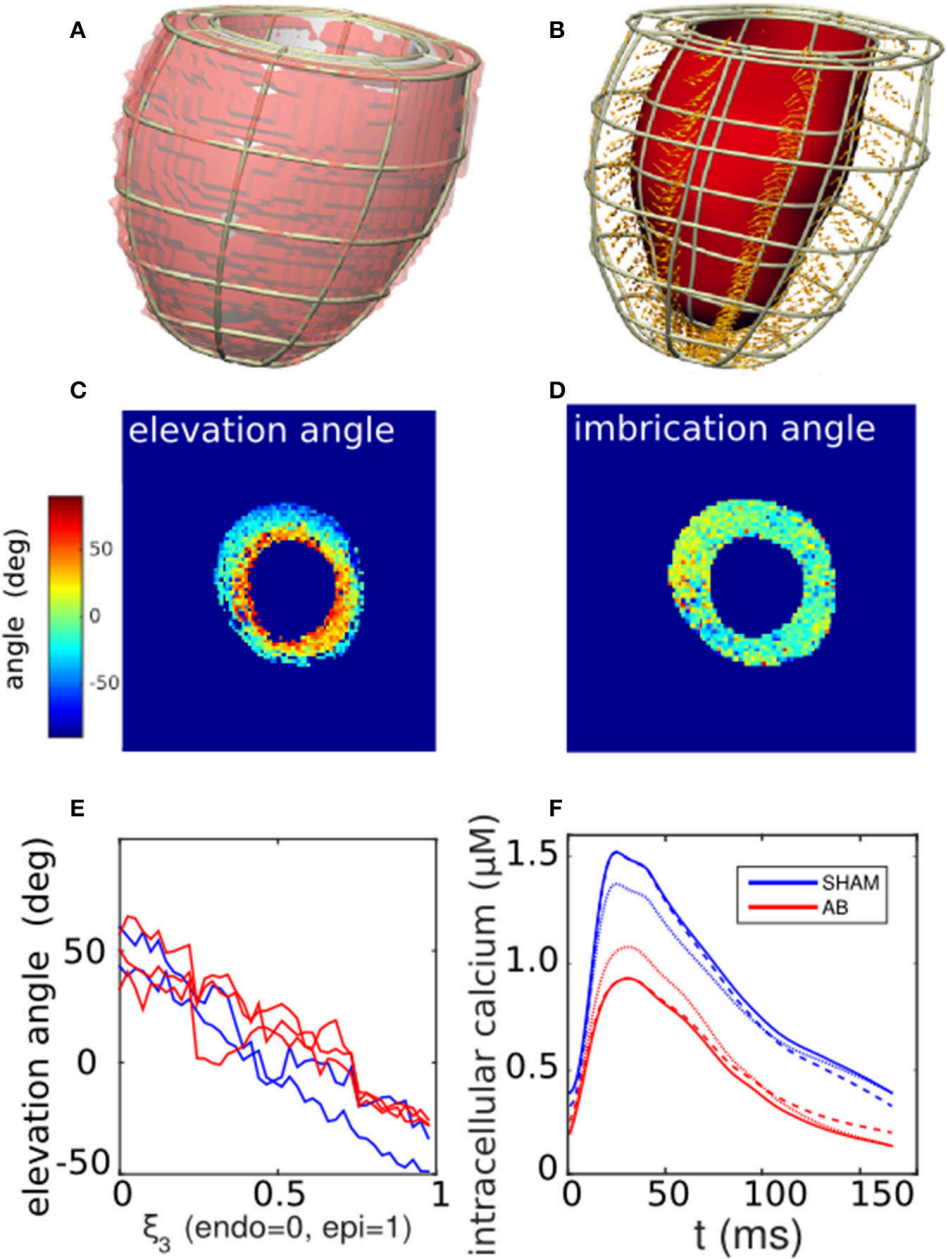
Cardiac tissue characterization. **(A)** Segmentation of a left-ventricle DT-MRI image (transparent red region) of a SHAM rat heart, and the corresponding fitted mesh volume (gray). **(B)** Corresponding finite-element mesh framework containing a selection of local fiber-orientation vectors. **(C,D)** Cross-sectional maps of the elevation (α) and imbrication angles (β) of the fiber vectors, calculated in relation to the local mesh basis vectors. Typically, α varies linearly from positive to negative values across the LV wall, while β shows little variation away from zero. **(E)** One-dimensional profiles of the average fiber elevation angle, derived from the DT-MRI analysis in **(C)** and repeated for available SHAM and AB hearts. Elevation angles are plotted as functions of normalized distance across the LV wall for the SHAM (blue traces) and AB hearts (red). **(F)** Comparison of measured calcium traces (solid lines) derived from SHAM and AB heart cells (section 2.5), and hypothetical “hybrid calcium traces” generated by a linear combination of the measured traces (section 2.6.3). The blue dashed curve represents the case 75% SHAM DCa and 25% AB DCa with 100% SHAM PCa, and *vice versa* for the red dashed curve. The blue dotted curve represents the case 75% SHAM PCa and 25% AB PCa with 100% SHAM DCa, and *vice versa* for the red dotted curve.

We limited the model to the LV, as no measurements were available to characterize right-ventricular funtion. The LV segmentation of individual rat hearts, derived from the DT-MRI images (ITK-SNAP Version 2.20), was fitted to a finite-element cubic-Hermite single-cavity mesh template comprising 6 elements along the longitudinal direction, 8 in the azimuth, and 2 across the wall thickness (Figures 3A,B) (Lamata et al., 2010, 2011, 2014). The fitting process involved minimizing the residual distance between the nodes of an LV template mesh and the endo- and epicardial surfaces of the three-dimensional image segmentation, while constraining the basal plane to be flat and perpendicular to the apex-base axis.

#### 2.6.2. Fiber configuration

The diffusion tensor in the muscle tissue was reconstructed from the DT-MRI images at each voxel contained within the mesh domain, taking the primary eigenvectors as specifying the fiber directions. The projection of each fiber vector onto the local geometrical basis vectors yielded the elevation (α) and imbrication (β) angles (LeGrice and Hunter, 1997). These two scalar fields were then incorporated into the mesh structure by cubic-Hermite fitting. The fiber directions defined the direction of active stress generation in the subsequent simulations.

#### 2.6.3. Electromechanical coupling

The cardiac cycle was simulated within the computational framework of Land et al. (Land and Niederer, 2011; Land et al., 2012). Briefly, an intracellular calcium stimulus was applied simultaneously throughout the ventricular tissue. The solid curves in Figure 3F, derived from measurements on SHAM- and AB-operated rat hearts (see section 2.5), were used to stimulate the corresponding SHAM and AB models. The traces are characterized by their peak (PCa) and diastolic levels (DCa). As explained in section 2.1, our sensitivity analysis (Equation 2) involved performing simulations using hypothetical transients, constructed from linear combinations of the measured traces with arbitrary PCa and DCa values. For instance, the dashed blue curve in Figure 3F has a DCa that is 75% SHAM and 25% AB with a purely SHAM PCa. An analogous hybridization of the PCa values is represented by the blue dotted curve.

The binding of calcium to troponin C, described by a Hill equation (Hill coefficient *n*_trpn_ = 2 and binding rate $k_{\text{trpn}}\;=\;0.1\text{ m}{\text{s}}^{-1}$), initiated the formation of myosin crossbridges that generate local active tension. For simplicity, the actin-myosin interaction was modeled as a two-state system (with proportions *f*_xb_ and 1 − *f*_xb_ of bound and unbound myosins), also governed by Hill dynamics (*n*_xb_ = 5, $k_{\text{xb}}=0.02\text{ m}{\text{s}}^{-1}$). The net active tension *T*_a_ generated for a given crossbridge fraction *f*_xb_ was modulated to reflect the length and stretching-velocity dependences as *T*_a_ = *T*_ref_*g*(*dλ*/*dt*; *t*)*h*(λ)*f*_xb_, assuming *T*_ref_ = 120 kPa as a scale prefactor consistent with experiments (Land et al., 2012). The function *h*(λ) expresses the length dependence for a local extension ratio λ, while *g* encapsulates the stretching-velocity dependence, based on a “fading memory” model (Hunter et al., 1998).

The passive mechanical behavior was governed via the transverse-isotropic strain-energy functional (Guccione et al., 1991)

(5) $$W=\frac{1}{2}c_{1}\exp\left[c_{2}E_{11}^{2}+c_{3}(E_{22}^{2}+E_{33}^{2}+2E_{23}^{2})+2c_{4}(E_{12}^{2}+E_{13}^{2}\right],$$

where *E* is the Lagrangian strain tensor expressed relative to the muscle fiber and sheet directions. The parameters *c*_2_ = 8.0, *c*_3_ = 2.0, and *c*_4_ = 3.7, which characterize tissue anisotropy, were based on mouse data (Omens et al., 1994), assumed to be the most accurate characterization for the rat. We used the prefactor *c*_1_ as the only free fitting parameter (see Figure 1 and section 2.8), treating it as a phenomenological scaling factor for the overall tissue stiffness. Only the prefactor *c*_1_ was varied to preserve the ratios between the different stiffness components.

#### 2.6.4. Computational method and boundary conditions

The mechanical solution method consists of solving the balance of forces subject to tissue incompressibility (Land et al., 2014). The spatial boundary conditions were defined by constraining the LV basal plane to lie perpendicular to the apex-base axis, with one mesh node on the interior LV wall fixed in all directions. Another node, located diametrically opposite on the LV interior wall, was constrained to move on the axis connecting the fixed node, thereby prohibiting rigid-body rotation.

The pressure boundary conditions were defined by the imposed aortic pressure *p*_a_, which is coupled to the cardiac system via a three-element Windkessel model (Figure 1). The Windkessel-model parameters were based on rat measurements (Westerhof and Elzinga, 1991): peripheral resistance *r* = 105 mmHg·s/ml, arterial compliance *c* = 0.014 ml/mmHg, and aortic impedance *z* = 6.3 mmHg·s/ml. AB was implemented by increasing *z* by 50% [*z*^(AB)^ = 9.5 mmHg·s/ml], keeping all other parameters unchanged.

### 2.7. Simulation protocol

The cardiac cycle was simulated using the protocol described in detail elsewhere (Land and Niederer, 2011; Land et al., 2012) to reproduce the heart cycle (pressure-volume loop in Figure 1). Briefly, a phenomenological model of diastolic filling governed LV inflation toward a set end-diastolic pressure of 1 kPa. Upon stimulating the muscle with a calcium transient, the LV volume was fixed to simulate isovolumetric contraction. The calcium transient, combined with the myocyte contraction model, generated active tension within the tissue along the fiber directions, resulting in a sharp LV pressure rise. Upon reaching the pre-set aortic pressure *p*_a_, the Windkessel model governed blood ejection from the LV until volume flow was reversed. Fixing the LV volume again in this state, isovolumetric relaxation was then initiated, terminating when the LV pressure attained the chosen diastolic pressure (1 kPa). Diastolic filling was then reactivated and the process was repeated until the pressure-volume loop converged to a steady-state limit cycle.

### 2.8. Parameter fitting

To minimize the reliance of the simulation on unmeasured parameters, we limited the analysis to consider a simplified phenotype space defined by three variables: the mean LV diameter at end diastole (LVD, measured from the echocardiography data), the maximum pressure *p*_max_ during LV ejection (obtained from the hemodynamic measurements), and EF (determined from the echo data using Equation 4). Each of these quantities can be compared directly with the simulation output (section 2.7).

Preliminary simulations were conducted to explore the parameter space. Variations in model parameters yielded smooth monotonic changes in the phenotypes, thereby justifying the use of a gradient-base method of solution. A Newton method was therefore used to fit each rat LV model, to reproduce the three corresponding phenotypes, in terms of three model parameters: (1) the effective tissue stiffness $\tilde{c_{1}}$, which predominantly constrains the end-diastolic volume; (2) the aortic pressure *p*_a_; and (3) the dissociation constant [Ca^2+^]_50_ between Ca^2+^ ions and troponin C, which controls the extent of the contraction (Land et al., 2012). Convergence of the solution was generally achieved after one or two iterations and the results showed no significant dependence on initial values.

In the absence of experimental data relating to diastolic filling, we assumed a constant diastolic pressure *p*_ed_ = 1 kPa throughout the filling phase. Consequently, the parameter $\tilde{c_{1}}$ encapsulates both the physical stiffness *c*_1_ (Equation 5) and the assumed *p*_ed_, i.e., $\tilde{c_{1}}\sim c_{1}/p_{\text{ed}}$.

## 3. Results

### 3.1. Echocardiography measurements

#### 3.1.1. Phenotype comparison

The absence of exterior symptoms of heart failure, in any of the rats considered, implies either that the AB had negligible effect on the hearts on the time scales considered, or that any effect of the AB was compensated by remodeling (Patten and Hall-Porter, 2009). To test the first hypothesis, we considered the diastolic wall thickness LVWT_dia_, a conventional marker of overload-induced concentric hypertrophy, calculated directly from the echo data, as illustrated in Figure 2A. To detect potential AB-induced hypertrophy, we compared the average relative rates of change *R*[LVWT_dia_] (see Equation 3) in the SHAM and AB hearts, rather than the final LVWT_dia_ values, to mitigate the natural variability observed between individual hearts.

The mean *R*[LVWT_dia_] value for the AB hearts of (10 ± 3)%/week (s.d.) is significantly greater than the corresponding SHAM rate (3 ± 2)%/week (*p* = 5 × 10^−5^) (Figure 2C). In contrast, having obtained EF using Equation (4), the mean *R*[EF] values for either cohort are not significantly different from zero (Figure 2D), with average values of 0.8%/week for the SHAM (s.d. = 2.2%/week, *n* = 10, *p* = 0.3) and −1.3%/week for the AB hearts (s.d. = 2.2%/week, *n* = 10, *p* = 0.1), suggesting that AB in these rat hearts had an insignificant effect on EF. This is further supported by the similarity in the mean EF values [0.75 ± 0.05 (s.d.), *n* = 10, for the SHAM hearts, vs. 0.71 ± 0.06 (s.d.), *n* = 10, for the AB hearts, *p* = 0.1], 4 weeks post intervention (Figure 2B). We therefore conclude that the systemic change driven by AB resulted in a clear increase in LV wall thickness while maintaining a constant EF at this stage of compensative remodeling in the rats considered.

#### 3.1.2. Model characterization

The echo measurements of specific rat hearts were used to parameterize the computational models for the simulations (section 2.1 and Figure 1). At *t* = 4 weeks, the echo data characterize the cardiac dynamics shortly before the hearts were extracted from the animals to perform the *ex vivo* DT-MRI analysis. This characterization allows the subsequent simulations to reproduce the *in vivo* contractions with maximum fidelity (section 2.8). Table 1 lists the diastolic and systolic LV diameters and EF values (*t* = 4 weeks) for these hearts.

**Table 1:** Measured phenotypes for the available SHAM and AB hearts.

**Heart**	**Measured**			
	**LVD**	**LVD**	**EF (%)**	***p*_max_ (kPa)**
	**diastole (mm)**	**systole (mm)**		
SHAM	8.1	4.9	70	17
AB1	7.9	4.6	72	25
AB2	8.6	5.3	70	19
AB3	7.5	4.3	73	28

The cardiac cycle is characterized by the diastolic and systolic LV diameters (LVD), the ejection fraction EF, and the maximum pressure *p*_max_ during ejection.

### 3.2. Geometry and fibers

Figure 3B shows a representative mesh constructed from an LV segmentation (Figure 3A). The fiber unit vectors, computed at each voxel contained within the mesh domain, were projected onto the local basis vectors of the mesh to yield elevation (α) and imbrication angles (β). The cross-sectional maps of α and β shown in Figures 3C,D are consistent with histological observations of fibers being predominantly parallel to the cavity walls (|β| ≲ 10°), and with α varying linearly from ~ +50° at the endocardial wall to ~ −40° at the epicardial (Figure 3E). For the purpose of the simulations, we therefore imposed β = 0 throughout the tissue, leaving α as a unique scalar field characterizing the fiber configuration. The fiber vectors were determined at the mesh nodes by cubic-Hermite fitting (Figure 3B).

### 3.3. Simulations

#### 3.3.1. Model fitting

Table 2 lists the fitted values for $\tilde{c_{1}}$, [Ca^2+^]_50_, and *p*_a_ that simulated the measured phenotypes with good accuracy (Table 1).

**Table 2:** Fitted model parameters and the resulting simulated phenotypes, which show good agreement with the corresponding measurements in Table 1.

**Heart**	**Model parameters**				**Simulated**			
	**$\tilde{c_{1}}$**	**[Ca^2+^]_50_**	***p*_a_**	**z**	**LVD diastole**	**LVD systole**	**EF**	***p*_max_**
	**(kPa)**	**(μM)**	**(kPa)**	**(mmHg·s/ml)**	**(mm)**	**(mm)**		**(kPa)**
SHAM	0.68	1.56	8.1	6.3	8.1	4.99	71	16.4
AB1	0.23	0.92	13.8	9.5	7.9	5.22	71	25.3
AB2	1.57	0.83	7.6	9.5	8.6	5.40	70	19.0
AB3	0.6	0.80	20	9.5	7.5	4.82	74	28.9

#### 3.3.2. Sensitivity analysis

**Figure 4:**
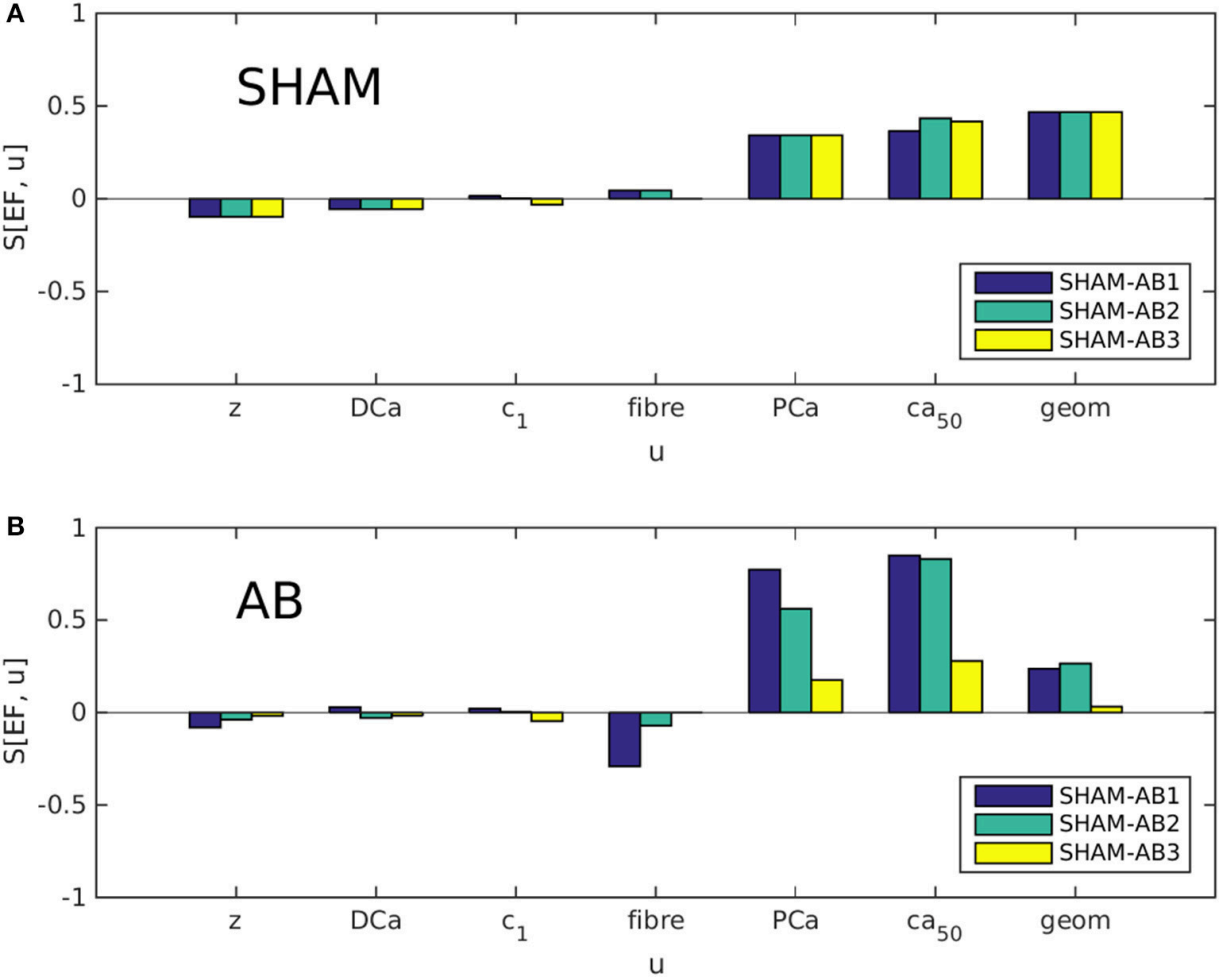
Partial sensitivities *S*[EF, *u*] of the ejection fraction EF, calculated with respect to each simulation parameter *u* using Equation 2. Each bar color denotes a different SHAM-AB heart pair, with *S*[EF, *u*] evaluated in the SHAM [**A**, *u* = *u*^(SHAM)^] and AB limits [**B**, *u* = *u*^(AB)^].

Partial sensitivities *S*[EF, *u*] were evaluated using Equation (2) for each coordinate in parameter space *U* (see Equation 1), in both the SHAM [*u* = *u*^(SHAM)^] and AB [*u* = *u*^(AB)^] cases. As explained in section 2.1, we examined three different trajectories in *U*, linking the available SHAM heart model to either of three AB models. Each bar in Figures 4A,B represents, in effect, the sensitivity to one variable *u* at one of these trajectory end points. Figure 4A indicates that the dominant sensitivities in the SHAM regime are those of PCa, [Ca^2+^]_50_, and geometry. In Figure 4B, however, the sensitivity associated with geometry is significantly reduced relative to the sensitivities to PCa and [Ca^2+^]_50_, for each heart pair. This change suggests a systematic decrease in the ability of hypertrophy to contribute toward the maintenance of the ejection fraction following aortic banding.

Although the direct effect of aortic banding is to increase the aortic impedance *z* artificially, *S*[EF, *z*] is relatively insignificant in both the SHAM and AB regimes, suggesting that the impact of the aortic constriction affects cardiac function only indirectly through cardiac remodeling. Similarly, *S*[EF, DCa], *S*[EF, $\tilde{c_{1}}$], and *S*[EF, *u*_fiber_] are consistently small. It is therefore unlikely that the large differences in parameter values between the AB hearts (see Table 2) have a significant influence on our present analysis.

**Figure 5:**
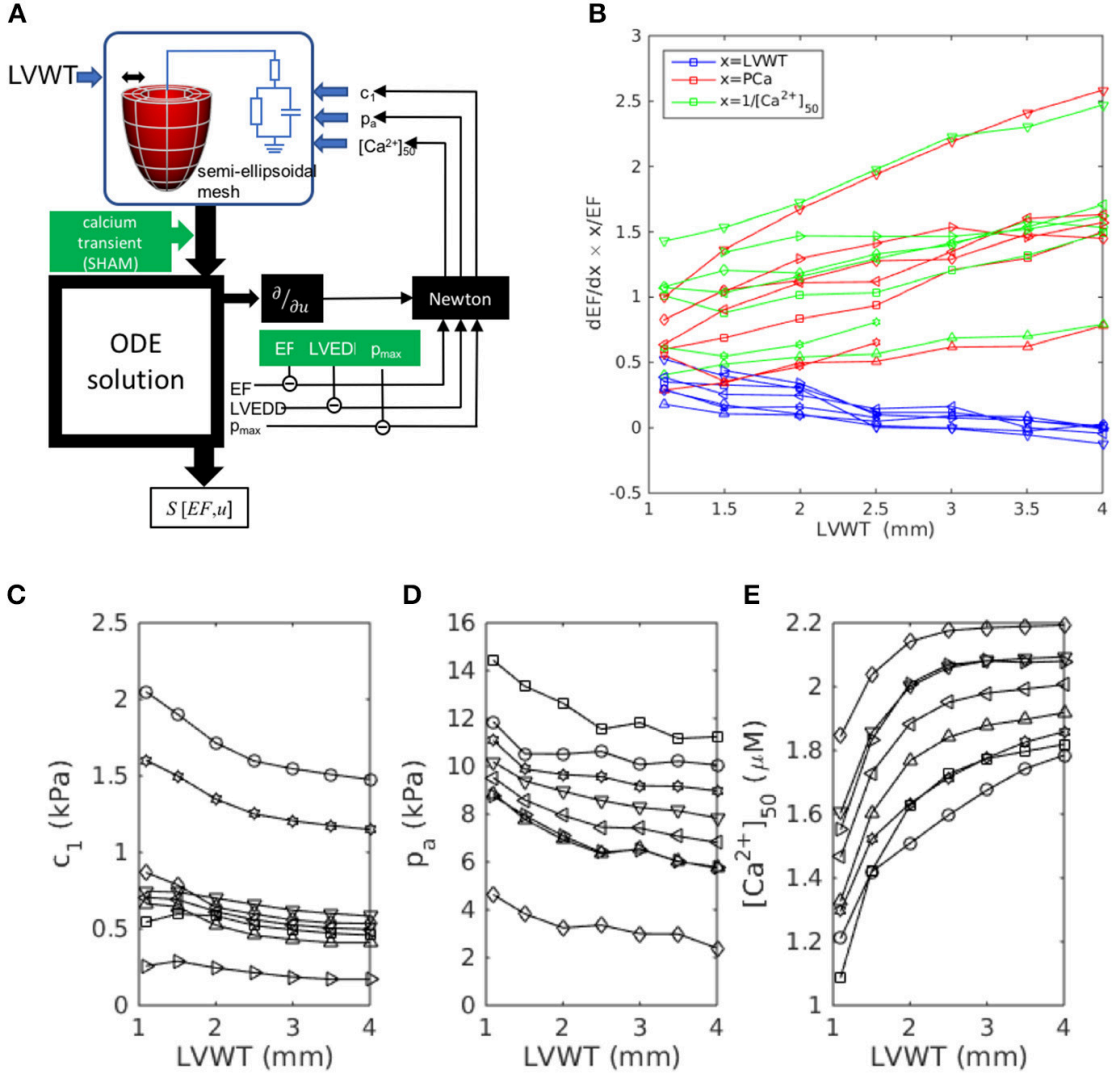
Wall-thickness dependence of the dominant sensitivities *S*[EF, *x*] identified in Figure 4, simulated using custom-designed canonical semi-ellipsoidal LV cavities (see section 3.3.2). **(A)** Workflow outline, adapted from Figure 1 for a semi-ellipsoidal mesh of arbitrary wall thickness LVWT. **(B)** Simulation results: sensitivities *S*[*EF, x*] computed for each target phenotype set: ⊳:(8.5 mm, 16 kPa, 75%), △:(7.5, 16, 75), ^*^:(9.0, 16, 75), °:(8.5, 20, 75), ▿:(8.5, 12, 75), □:(8.5, 16, 65), ◇:(8.5, 16, 85). **(C–E)** The model parameters *c*_1_, *p*_a_, and [Ca^2+^]_50_ were determined by Newton fitting to yield the target phenotypes for each LVWT.

To test the hypothesis that this evolution results from the increase in LV wall thickness LVWT specifically, we repeated the simulations, this time using canonical semi-ellipsoidal heart meshes, constructed with arbitrary LVWT, instead of the meshes derived from actual hearts. The analysis procedure is outlined in Figure 5A. By adopting the same mesh topology as the data-fitted meshes, and applying the same fiber elevation angle as for the real SHAM hearts ($\alpha_{\text{endo}}=+50^{{}^{\circ}}$, $\alpha_{\text{epi}}=-50^{{}^{\circ}}$, see Figure 3E), these custom-designed meshes allowed the isolation of LVWT as an independent geometrical variable. The simulations were repeated to cover a range of phenotype values (LVEDD, *p*_max_, EF) that straddled the measured SHAM phenotype (Table 1), i.e., 7.5 mm < LVEDD < 9.0 mm, 12 kPa < *p*_max_ < 20 kPa, and 65% < EF < 85%. For each chosen set of phenotype values, semi-ellipsoidal meshes were constructed with a range of LVWT, a constant cavity length of 10 mm, and a reference diameter of 3 mm. In each case, *c*_1_, *p*_a_, and [Ca^2+^]_50_ were refitted using the same Newton method described in section 2.8 to yield the target LVEDD, *p*_max_, and EF. All other model parameters, including the intracellular calcium transient, were the same as those used previously for the SHAM heart, and the fiber orientations in the reference meshes were set to vary linearly across the wall from α = +50° (endo) to −50° (epi). The fitted values are plotted in Figures 5C–E as functions of LVWT for each set of phenotype values considered.

Figure 5B plots the sensitivities *S*[EF, LVWT], *S*[EF, PCa], and *S*[EF, [Ca^2+^]_50_] as functions of LVWT. In all the cases, *S*[EF, LVWT] decreases with increasing LVWT, while *S*[EF, PCa] and *S*[EF, [Ca^2+^]_50_] both increase, mimicking the trends observed in Figure 4. This qualitative behavior was further shown to be insensitive to the fiber-orientation profile (e.g., $\alpha_{\text{endo}}=80^{{}^{\circ}}$, $\alpha_{\text{epi}}=-60^{{}^{\circ}}$), confirming that the observed effect was not limited to one specific heart. A notable feature of Figure 5B is the similarity in the gradients of *S*[EF, PCa] and *S*[EF, [Ca^2+^]_50_], despite the changes in the absolute values of [Ca^2+^]_50_ required to reproduce the phenotypes (Figure 5E) for a constant calcium stimulus. We note that, whereas the *U*-space trajectory from the SHAM to the AB hearts indicated an increase in calcium sensitivity (decreasing [Ca^2+^]_50_), the opposite tendency is observed here: [Ca^2+^]_50_ must increase with LVWT to yield the correct phenotypes. Yet, *S*[EF, [Ca^2+^]_50_] mirrors *S*[EF, PCa] in the same manner as in Figure 4. Further simulations were performed to compare the effect of varying the calcium-transient amplitude (Appendix 2). The increase in *S*[EF, PCa] with increasing LVWT is again observed, albeit at a smaller rate when applying the AB—rather than the SHAM—calcium transient. These results, taken together, emphasize the particular role played by LVWT in governing the qualitative evolution in compensatory properties.

## 4. Discussion

The aim of the present study was, firstly, to apply a data-driven model of the rat heart that reproduces the principal mechanisms by which the heart can compensate for changes in its environmental conditions to preserve its physiological function. Secondly, we sought to identify how this compensatory capacity may evolve in rats as a result of aortic-banding-induced ventricular pressure overload, which is known to instigate cardiac remodeling on various spatial scales. This was done by integrating electrophysiological, echocardiography, and DT-MRI measurements into heart-specific computational models, to allow the isolation and comparison of individual compensatory effects. The models, constructed using the available data, aimed to be as rat-specific as possible to reproduce the measured phenotypes. The variation in model parameters between the different hearts is comparable to variations reported in other studies. For example, Borbély et al. (2005) report standard deviations of up to 33% in cardiac tissue stiffness, a value not inconsistent with our fitted values.

We considered three broad classes of cardiac properties: the heart geometry (characterized implicitly by the LV cavity volume and the LV wall thickness), the muscle-fiber configuration, and the intracellular electrophysiological mechanisms, the output of which is the calcium transient that initiates muscle contraction. Although none of the examined rats displayed exterior symptoms of heart failure at the time of the measurement (as displayed by their constant EF), those rat hearts that had been subjected to aortic banding showed clear signs of LV-wall hypertrophy, indicative of the compensatory phase of cardiac remodeling. Intracellular calcium transients measured in LV myocytes derived from aortic-banded rats, albeit of a different breed, also showed significant remodeling. Further, we found that despite these changes, the EF was conserved following aortic banding. Using Equation (2), we therefore quantified the compensatory ability, in the aortic-banded and control hearts, in terms of the partial sensitivities of the EF to individual cardiac parameters. We interpreted these sensitivities as reflecting the capacity of the cardiac system to adapt to changes in physiological conditions in order to maintain its function, as characterized by the EF.

### 4.1. Evolution of compensation mechanisms

Our main results, summarized in Figure 4, identify an evolution in the subset of cardiac properties with the greatest ability to regulate EF. Whereas the EF sensitivities associated with the LV geometry and tension generation (via Ca^2+^ binding to troponin C) are of similar magnitude in the hearts with no aortic banding, the latter sensitivities increase significantly at the expense of the former following aortic banding. In other words, a healthy heart can be expected to compensate better for the onset of hypertension by a thickening of its cavity wall, than a heart in an advanced state of concentric hypertrophy; at that stage, remodeling of calcium signaling becomes a more effective compensatory mechanism. The complementary simulations summarized in Figure 5 and Appendix 2 further identify the wall thickness as the variable that most significantly governs the evolution in compensatory ability observed in Figure 4. The consistency of the observed behavior, over a range of phenotype values, supports the generality of the effect, beyond the few experimental cases available in the present study.

Another natural concern for this interpretation is that the hypertrophic process necessarily occurs much more slowly than the sudden impact of aortic banding. However, our simulations probed only the experimentally accessible end points of the overall remodeling process. Therefore, insofar as aortic banding serves as an experimental model for the more gradual onset of hypertension, our interpretation remains independent of the evolution of the rat hearts between these end points.

Concentric hypertrophy, clearly exhibited in the wall thickening of the AB hearts, has been interpreted as a passive compensatory response to pressure overload, insofar as it reduces the wall stress in the presence of enhanced LV pressure (Laplace's law) (Grossman et al., 1975; Olivetti et al., 1988). Its supercession by remodeling of the calcium dynamics, through PCa and [Ca^2+^]_50_, 6 weeks after the intervention, marks a qualitative transition in the heart's capacity to achieve compensation. The peak calcium level PCa encapsulates the underlying electrophysiological machinery of the muscle cells, which expends energy at every heart beat. The increase in *S*[EF, PCa] in the AB hearts can be explained by the lowering of PCa (Figure 3F). In other words, a given absolute change in calcium becomes proportionally more significant in an AB than in a SHAM heart. Our dynamic model fits indicate a concomitant decrease in [Ca^2+^]_50_, the intracellular Ca^2+^ concentration required to produce 50% occupancy of the troponin C regulators of actomyosin cross-bridge formation. Biochemically, [Ca^2+^]_50_ is controlled by the phosphorylation of troponin I through various kinases (Layland et al., 2005). There was no *a priori* reason for the [Ca^2+^]_50_ values listed in Table 2 to vary in the same manner as the measured PCa values (1.53 μM for SHAM-heart cells, 0.93 μM for AB). The model fitting, however, implies a strong correlation between those parameters that arguably reflects a functional benefit by maintaining contraction dynamics near the middle of its dynamic range.

The observed depression of the Ca^2+^ transient in cases of heart failure has been associated with impaired contractile behavior (Siri et al., 1991). However, when accompanied by a proportional decrease in [Ca^2+^]_50_, this change may benefit compensation by magnifying the impact of small changes in the calcium concentration on EF. This evolution may become increasingly beneficial with the decline of compensation through hypertrophy.

### 4.2. Model simplification

By limiting the scope of our simulations to the principal features of diastole and systole, we reduced the parameter space to maximize the objectivity of the model parameterization. We lumped the passive mechanical tissue properties into the single phenomenological parameter $\tilde{c_{1}}$, amalgamating the physical stiffness *c*_1_ and the unknown end-diastolic pressure *p*_ed_. The measured end-diastolic diameter was hence uniquely fitted. In systole, tension generation is controlled by both the intracellular calcium dynamics and the strength of crossbridge contraction. Aortic banding can in principle affect either—or both—of these contributions. As outlined in section 2.6.3, contractile strength is itself influenced by several mechanisms, whose detailed characterization lies outside the scope of the available measurements. We therefore chose to lump the sensitivity to tensile behavior in the calcium-binding parameter [Ca^2+^]_50_, and assumed a constant *T*_ref_ in all the hearts. This simplification assumes that an impact on *T*_ref_ by aortic banding would likely involve a substantial remodeling of the myofibril structure. On the other hand, a change in [Ca^2+^]_50_ through the modified expression of kinases arguably involves a simpler regulation, at the protein level, that can more reasonably be expected to occur in the early stages of hypertrophy. Variations in calcium sensitivity in cases of heart failure have been reported, as a result of the phosphorylation of myofibrillar regulatory proteins (Wolff et al., 1995; Velden et al., 2009).

### 4.3. Limitations

DT-MRI is a costly and delicate technique that affords a considerably lower throughput than echocardiography. Thus, whereas the latter experiments provided statistically significant samples of geometric measurements, only a small proportion of those hearts underwent DT-MRI processing. As explained above, the main appeal of performing both experiments successively on the same hearts was to increase the consistency of the models with the biological systems. One performance criterion for the computational simulations was their ability to reproduce the morphological dynamics inferred from the echocardiograms (see section 2.8). Of the initial cohort of hearts (ten SHAM and ten AB), two SHAM and four AB hearts proceeded to DT-MRI. Within this subgroup, only one SHAM heart and three AB hearts successfully simulated the expected diastolic size and EF. In both of the unsuccessuful cases, blood ejection into the aorta stalled at a fraction of the expected EF, even when the strength of contraction (*T*_ref_) was increased in the extreme. We deduced that this failure to achieve the expected dynamics resulted from the nominal LV size in diastasis (derived from the DT-MRI measurements) being almost identical to the diastolic diameter (to within 10% or less). In contrast, the successfully simulated hearts had an equilibrium size that typically amounted to 60–70% of the end-diastolic volume, which provided scope for sufficient contraction. One potential cause of error in the erroneous DT-MRI measurement is the possibility of even a small intramyocardial pressure being exerted during perfusion with the high-K^+^ arrest solution and fixative. The near coincidence of the LV diameter in this state with the measured end-diastolic size is consistent with an unintended inflation of the LV. We therefore considered these anomalies as artifacts and discarded those hearts from the analysis, retaining one SHAM and three AB hearts.

The model characterizations would no doubt have benefitted from additional measurements, for example of tissue deformation by MRI or echography. However, these measurements were not part of the initial experimental protocol. Although future exploration of these parameters would be desirable, we evaluated LV torsion in the simulated contractions (angle of twist per unit length of ventricular wall) and found no systematic trend distinguishing the SHAM and AB hearts. The available measurements thus provide no evidence that this is a significant parameter for the present purpose.

To increase confidence in the validity of our conclusions despite the paucity of available hearts, the complementary simulations of Figure 5 and Appendix 2, done with the canonical semi-ellipsoidal heart meshes, explored a range of the phenotype space around the SHAM heart (see Table 1: SHAM). All the cases considered display the same qualitative behavior, with the sensitivity *S*[EF, LVWT] decreasing with increasing LVWT while *S*[EF, PCa] and *S*[EF, [Ca^2+^]_50_] both increasing. Taken together, these results demonstrate the general pattern that, as LVWT increases, EF becomes less sensitive to variations in LVWT and more sensitive to those of [Ca^2+^]_50_ and PCa.

### 4.4. Conclusion

To our knowledge, the present work constitutes a first attempt to provide a global view of cardiac compensation, within the context of ventricular pressure overload, by considering the anatomical shape and the passive and active mechanical behavior on an equal footing. We conclude that intracellular behavior becomes a more efficient compensatory mechanism than organ-level remodeling as the rat heart responds to the onset of pressure overload. This phenomenon was observed with regard to EF, a traditional indicator of cardiac dysfunction routinely measured in clinical protocols. Although other metrics of cardiac function (e.g., cardiac output and the stroke volume per body weight) directly reflect cardiac efficacy, those metrics are notoriously dependent on temporal and biological factors, and may vary significantly among healthy subjects of the same species, or even, over time, in a given individual (Haxhe and Lammerant, 1964; Ovsyshcher et al., 1993; Collis et al., 2001). Such incidental influences are arguably normalized in EF, allowing a more equitable comparison of different hearts. Furthermore, the EF is preserved at its healthy level in nearly half of heart-failure patients (Sharma and Kass, 2014). One major obstacle in fully understanding this increasingly prevalent condition is the limitation of existing experimental models for highlighting the distinct facets of the disease. Toward meeting this challenge, computational modeling provides an invaluable basis for unifying experimental results and investigating subsystems within an integrative perspective.

## Author contributions

AL performed the computational analysis, analyzed the experimental data, wrote most of the manuscript. SL wrote the software code for the computational simulations. EC, LF, JO, and AM provided the MRI images of the rat hearts. PL wrote the software code for creating the finite-element meshes used for the simulations. SN and NS contributed significantly to the conceptual design of the work, the analysis of the results and the writing of the manuscript.

### Conflict of interest statement

AM and JO are co-founders, equity holders and scientific advisors to Insilicomed Inc., a licensee of UCSD software used in this research. This relationship has been disclosed to the University of California San Diego and is overseen by an independent conflict of interest management subcommittee appointed by the university. The other authors declare that the research was conducted in the absence of any commercial or financial relationships that could be construed as a potential conflict of interest.
