# Supplementary material for: Decreasing Compensatory Ability of Concentric Ventricular Hypertrophy in Aortic-Banded Rat Hearts
Source: Front Physiol. 2018 Feb 23;9:37. doi: 10.3389/fphys.2018.00037 (PMC5829063; doi:10.3389/fphys.2018.00037)
Supplement: Supplementary file 1 [file DataSheet1.pdf]

SUPPLEMENTARY MATERIAL: Decreasing compensatory ability of concentric ventricular hypertrophy in aortic-banded rat hearts

Alexandre Lewalle [1], Sander Land [1], Eric Carruth [2], Lawrence R Frank [3], Pablo Lamata [1], Jeffrey H Omens [2,4], Andrew D McCulloch [2,4], Steven A Niederer [1], Nicolas P Smith [5,*]

[1] *Department of Biomedical Engineering, King's College London, St. Thomas's Hospital, London SE1 7EH, United Kingdom*
[2] *Department of Bioengineering, UC San Diego*
[3] *Radiology Department, UC San Diego*
[4] *Department of Medicine, UC San Diego, 9500 Gilman Drive, La Jolla, CA 92093, USA*
[5] *Faculty of Engineering, University of Auckland, 70 Symonds Street, Auckland, New Zealand*

Correspondence*:
Corresponding Author
np.smith@auckland.ac.nz

APPENDIX 1 - VALIDATION OF EF MEASUREMENTS FROM M-MODE ECHOCARDIOGRAMS

The formula used to calculate the LV ejection fraction from the M-mode echocardiograms (Equation 4) effectively models the LV as a cylindrical cavity with a wall of constant volume. Within this model, the ejection fraction determined from the variation of the total cylinder volume over a full heart beat is identical to the ejection fraction corresponding to an arbitrarily thin cylindrical slice extracted from the large cylinder. The M-mode echo images provide the cavity diameter $2r$ and wall thickness LVWT of such a slice, from which the relative slice-thickness variation can be deduced by assuming a constant tissue volume.

To validate this approach, we tested Equation (4) using LV volume variations in rat hearts measured using MRI by (Wise et al., 1998). **Figure A1(a)** shows the variation, over a heart beat, in the longitudinal cross section through the LV (image reproduced from Figure 8 of (Wise et al., 1998)). To mimick the M-mode echo measurement on these MRI-based images (see **Figure 2A**), we cut three horizontal slices through the middle region of the LV and identified their intersections with the cavity wall. For each time point and each slice, we obtained the LV cross-sectional area $A = \pi r^2$ and LV wall thickness LVWT, giving a slice thickness

$$h \sim \frac{\mathrm{const}}{\pi(r + \mathrm{LVWT})^2 - \pi r^2}, \tag{A1}$$

thereby guaranteeing a constant tissue volume. The internal cavity volume $V \sim Ah$ is plotted in Figure A1(b) for each of the three slices (dashed lines). The average volume (solid curve) yields an ejection fraction $\mathrm{EF} = 1 - V_{\min}/V_{\max}$ of $(67 \pm 3)\%$, in good agreement with the $(64 \pm 3)\%$ computed by (Wise et al., 1998) by integrating MRI-based cross sections over the entire LV volume. We therefore conclude that the EF estimates derived from the M-mode echo measurements are sufficiently accurate to allow comparisons between the rat hearts considered in the present study.

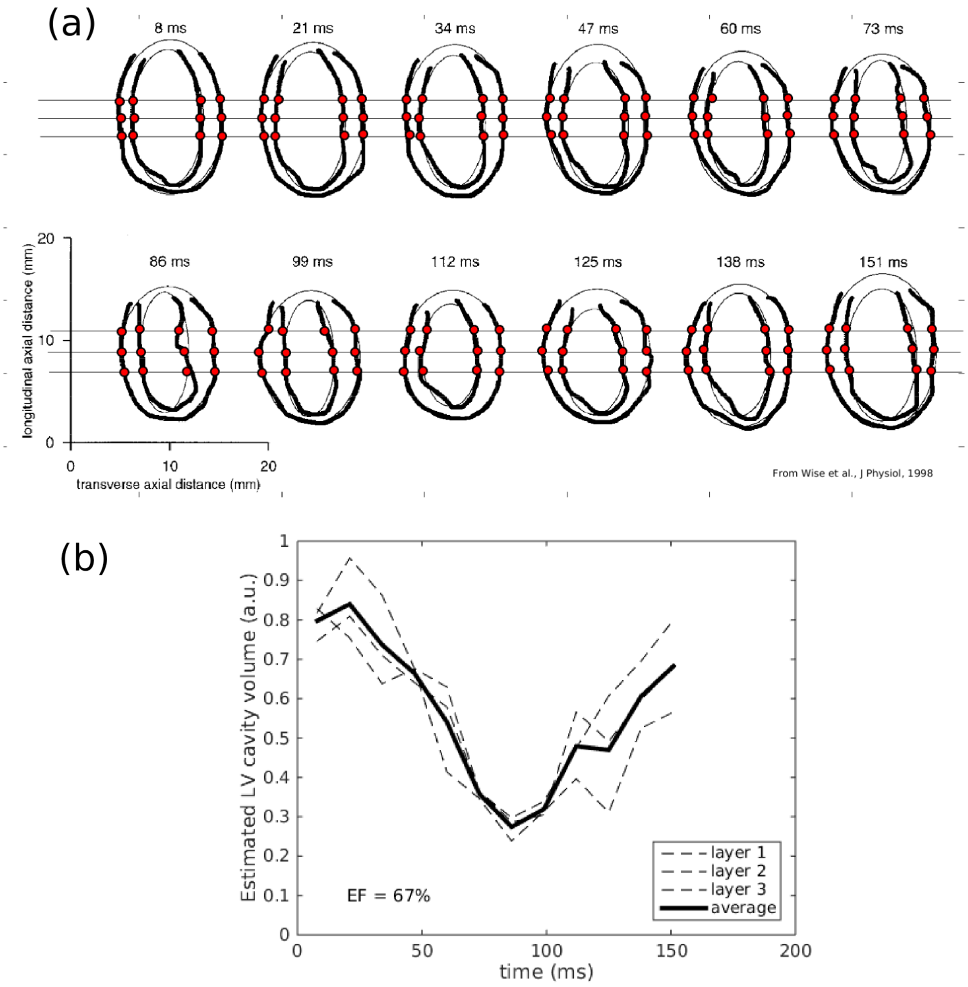

Figure A1. **(a)** Cross-sectional profiles of a rat LV cavity sampled at 13 ms intervals over one heart beat, constructed from MRI images (taken from Figure 8 of Wise et al. (1998). The red dots (our addition) are the intersections of the cavity wall surfaces with three horizontal sections drawn (by us) through the middle of the cavity. **(b)** Dashed lines: LV volume variations, estimated for each layer in **(a)** using Equation 4 ; solid line: average volume variation. The estimated EF is $(67 \pm 3)\%$.

APPENDIX 2 - SENSITIVITY TO THE CALCIUM AMPLITUDE

The simulations of **Figure 5** , performed on canonical semi-ellipsoidal LV meshes of varying wall thickness but with a constant calcium transient, confirm the trend displayed in **Figure 4** : the increase in wall thickness LVWT, which characterises concentric LV hypertropy, is associated with a decrease in the ability of LVWT itself to maintain EF at its constant value. To assess the contribution to the EF sensitivities of the calcium transient magnitude (another feature of the SHAM-AB transition), simulations were repeated using the calcium transients associated with the SHAM and AB hearts.

As in the earlier simulations, the model parameters c_1, p_a, and $[\mathrm{Ca}^{2+}]_{50}$ were adjusted so as to yield set values for the heart-cycle phenotypes LVEDD, $p_{\max}$, and EF. The results, compared in **Figure A2** , confirm the previously observed trend, namely that the sensitivity $S[\mathrm{EF}, \mathrm{LVWT}]$ decreases with increasing LVWT. In addition, although $S[\mathrm{EF}, \mathrm{PCa}]$ and $S[\mathrm{EF}, [\mathrm{Ca}^{2+}]_{50}]$ both increase, the rate of increase of $S[\mathrm{EF}, \mathrm{PCa}]$ is lower for the AB than for the SHAM calcium transient. The results suggest that, although both anatomical and intracellular properties contribute to the compensatory ability of the heart (as quantified by the sensitivites $S[\mathrm{EF}]$, the growing significance of PCa as a compensatory mechanism arises from the increase in the anatomical wall thickness.

REFERENCES

Wise RG, Huang CH, Gresham GA, Al-Shafei AIM, Carpenter TA, Hall LD. Magnetic resonance imaging analysis of left ventricular function in normal and spontaneously hypertensive rats. *Journal of Physiology* **513** (1998) 873–887.

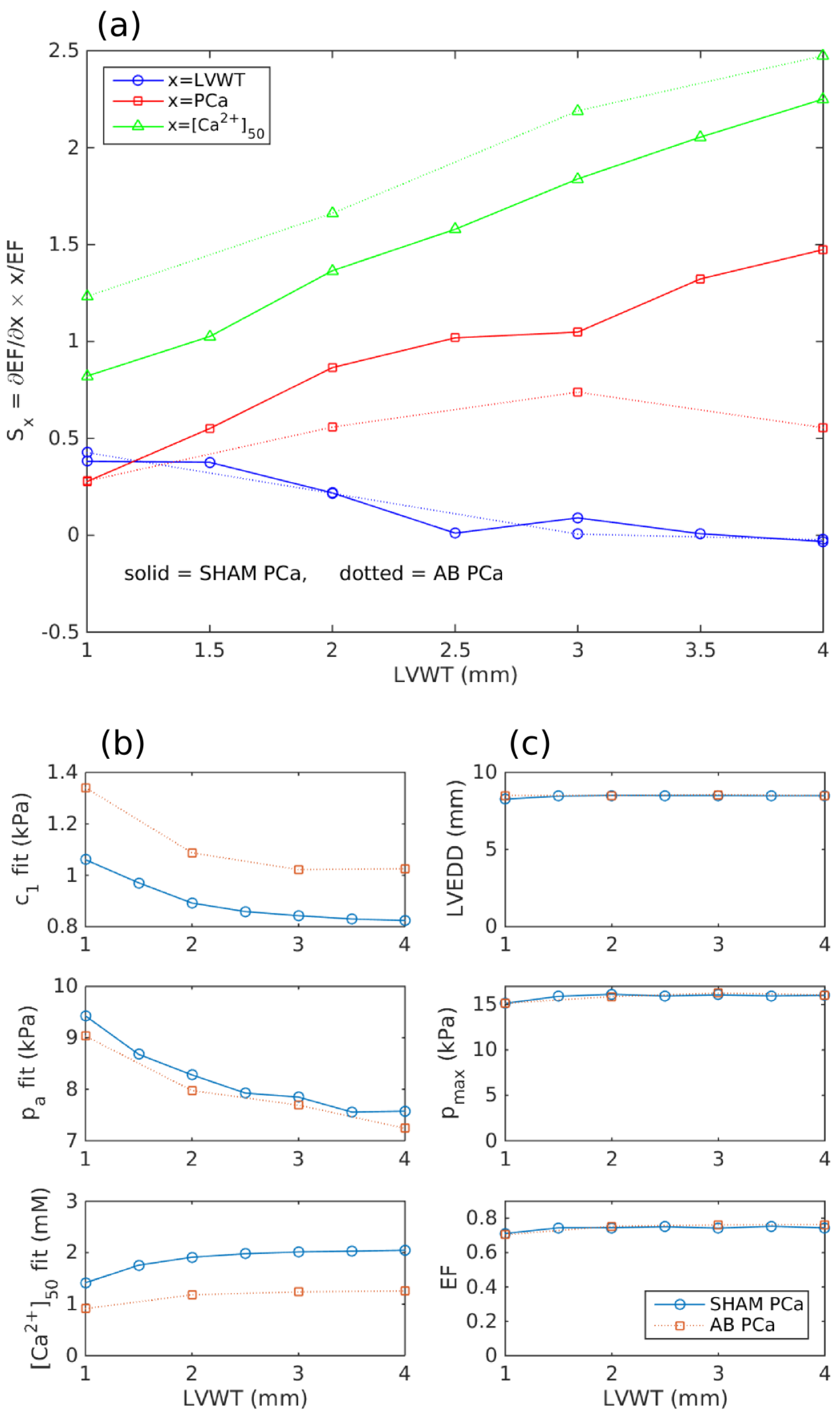

Figure A2. **(a)** Wall-thickness dependence of the sensitivities $S[\mathrm{EF}, x]$ (with x = wall thickness WT, peak calcium PCa, and the calcium binding affinity $[\mathrm{Ca}^{2+}]_{50}$) in semi-ellipsoidal LV cavities stimulated with the SHAM (solid curves) or AB calcium transients (dotted curves). For each WT value and for each calcium transient, the model parameters **(b)** c_1, p_a, and $[\mathrm{Ca}^{2+}]_{50}$ were determined (see workflow in **Figure 5A**) to yield constant values for the phenotypes **(c)** LVEDD, p_max, and EF.
